# Primary Immunodeficiencies in a Mesoregion of São Paulo, Brazil: Epidemiologic, Clinical, and Geospatial Approach

**DOI:** 10.3389/fimmu.2020.00862

**Published:** 2020-05-12

**Authors:** Denise Helena Boton Pereira, Lívia Souza Primo, Giovana Pelizari, Edilson Flores, Dewton de Moraes-Vasconcelos, Antonio Condino-Neto, Luiz Euribel Prestes-Carneiro

**Affiliations:** ^1^Imunnodeficiencies Outpatient Clinic, Regional Hospital of Presidente Prudente, Presidente Prudente, Brazil; ^2^Department of Pediatrics, Oeste Paulista University, Presidente Prudente, Brazil; ^3^Department of Statistics, Paulista State University, Presidente Prudente, Brazil; ^4^Laboratory of Medical Investigation Unit 56, Hospital das Clínicas da Faculdade de Medicina da Universidade de São Paulo, São Paulo, Brazil; ^5^Department of Immunology, Institute of Biomedical Sciences, University of São Paulo, São Paulo, Brazil; ^6^Department of Internal Medicine, Oeste Paulista University, Presidente Prudente, Brazil

**Keywords:** primary immunodeficiencies, active search, antibody deficiency, autoimmune diseases, mesoregion, epidemiology, geospatial

## Abstract

**Background:** Primary immunodeficiencies (PIDs) are rare genetic disorders leading to immunologic abnormalities that can affect different organs and systems. We determined the epidemiology, clinical, and geospatial characteristics of PID disorders among patients diagnosed over a 5 year period in a reference hospital covering a mesoregion in São Paulo, Brazil.

**Methods:** A retrospective analysis of 39 patients with recognizable PIDs according to the criteria of the European Society of Primary Immunodeficiencies were enrolled. Thirty-four patients came from outpatient immunodeficiency clinics and five patients from active search. Demographic, clinical, and immunologic data were collected, and maps were constructed using a geographic information system.

**Results:** The ratio of females to males was 1.4:1, and 48.7% of patients were younger than 17 years of age. The mean age at the onset of symptoms in children was 2.0 years [standard error of the mean (SEM), 1.7 years] and the diagnosis lag was 5.1 years (SEM, 3.1 years); the mean age at diagnosis in adults was 16.3 years (SEM, 11.8 years) and the lag was 10.8 years (SEM, 10.9 years). Antibody deficiency and common variable immunodeficiencies were the most common categories and phenotypes, respectively. The need for intravenous antibiotics and respiratory tract infections were the most prevalent warning signs, with an overall mortality rate of 15.3%. Autoimmune diseases were diagnosed in 56.4% and visceral leishmaniasis in 5.1% of patients. In the active search, 29 patients were investigated and 17.2% were diagnosed; early diagnosis, the involvement of multidisciplinary professionals, and dissemination of knowledge achieved milestone benefits. The distribution of PID networks in Brazil shows great asymmetry between regions and at a regional level; it was shown that the patients lived mainly in Presidente Prudente municipality.

**Conclusions:** The implementation of an immunodeficiency outpatient clinic in a referral hospital covering a mesoregion with a large population has led to the generation of policies and practices to improve the diagnosis, quality of life, and care of patients with PIDs and their families. Furthermore, the search for hospitalized patients with warning signs for PIDs showed great benefits. Inequality in the distribution of PID network centers in Brazil was demonstrated.

## Introduction

Primary immunodeficiencies (PIDs) comprise a heterogeneous group of genetic disorders, leading to immunologic abnormalities that can affect different organs and systems. They are caused by genetic defects and can arise early, at birth or during childhood or even adulthood, affecting all age groups (1–4). They have a broad phenotype with increased morbidity and mortality, and treatment choices are often complex (2–4). One of the main characteristics of PIDs is a reduction in the individual's ability to resist bacterial, fungal, parasitic, and severe viral infections. In addition, these patients can present non-infectious manifestations, such as autoimmune diseases, allergies, and increased vulnerability to tumors (1, 4).

Recent studies have shown that PIDs may be more common than previously estimated and that as many as 1% of the population may be affected (5). Published data on PIDs in Brazil are scarce, and the prevalence is not known. Furthermore, medical professionals are often not fully informed about the clinical presentation of PIDs, and patients remain undiagnosed or untreated for several years (6).

Spatial analytic techniques have emerged as innovative and important tools in public health and epidemiologic studies. They can add a great deal to the research on rare diseases such as PIDs and cancers by exploring the locations where cases are recorded, data management, and visualization processes (7). The aim of this study was to determine the epidemiologic, clinical, and geospatial characteristics of PID disorders among patients diagnosed over a 5 year period in a reference hospital that covers a mesoregion in São Paulo, Brazil.

## Materials and Methods

### Study Design, Methods, and Patient Enrollment

**Figure 1:**
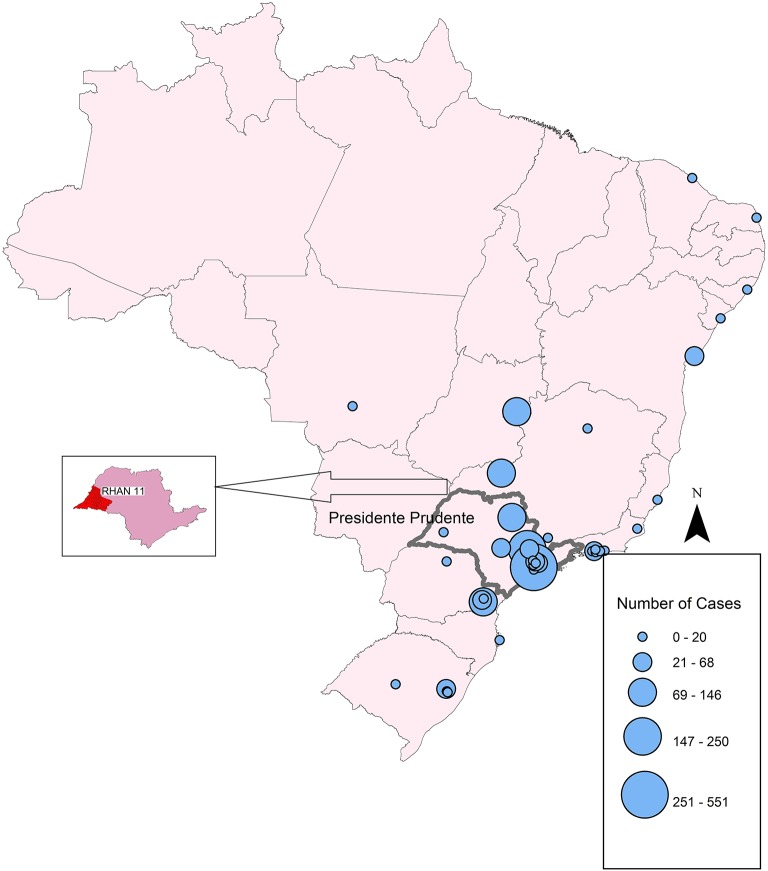
Map of the geographic distribution of PID network centers in Brazil, showing the concentration in São Paulo State. Circles represent the number of registered patients in 2018.

**Figure 2:**
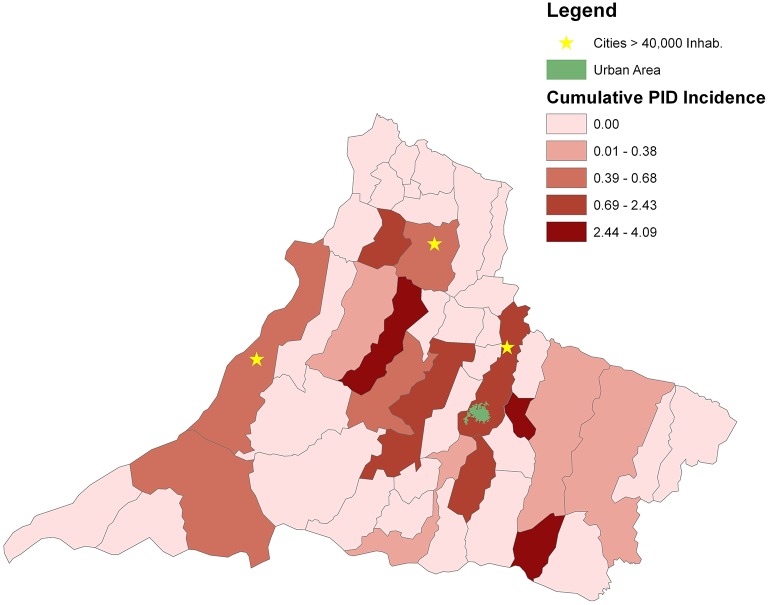
Geospatial distributions and cumulative incidence for patients with a PID diagnosis in the counties of RHAN.

In 2019, São Paulo, the richest and most populous state of Brazil, with an estimated population of 45,919,049 inhabitants, accounts for 21.8% of the entire population of Brazil, estimated to be 210,147,125 inhabitants, according to the Brazilian Institute of Geography and Statistics (IBGE; Figure 1). Geographically, the state is divided into 15 mesoregions and into 18 Regional Health Care Networks (RHCN) for administrative health care. The western region, with 45 municipalities and an estimated population of 753,344 inhabitants (2018), comprises the Regional Health Assistance Network11 (RHAN), located in Presidente Prudente mesoregion 8. The city is a mid-sized urban center 560 km from the state capital, São Paulo. According to IBGE, in 2019, the estimated population was 228,743 inhabitants (Figure 2).

The Regional Hospital of Presidente Prudente (RH) is a 550-bed, tertiary, public, and university hospital for RHAN, and in 2018, 23,104 patients were hospitalized at a monthly rate of 1,925 ± 1,142 patients. The hospital also receives a high number of patients in outpatient clinics covering 30 specialties. In 2018, 171,396 patients attended at a monthly rate of 14,283 ± 1,950 patients. In January 2014, the outpatient immunodeficiency clinic was created to attend to patients suspected of having PID referred from other clinics, including dermatology, hematology, pneumology, pediatrics, infectious diseases, gastroenterology, rheumatology, and oncology.

**Figure 3:**
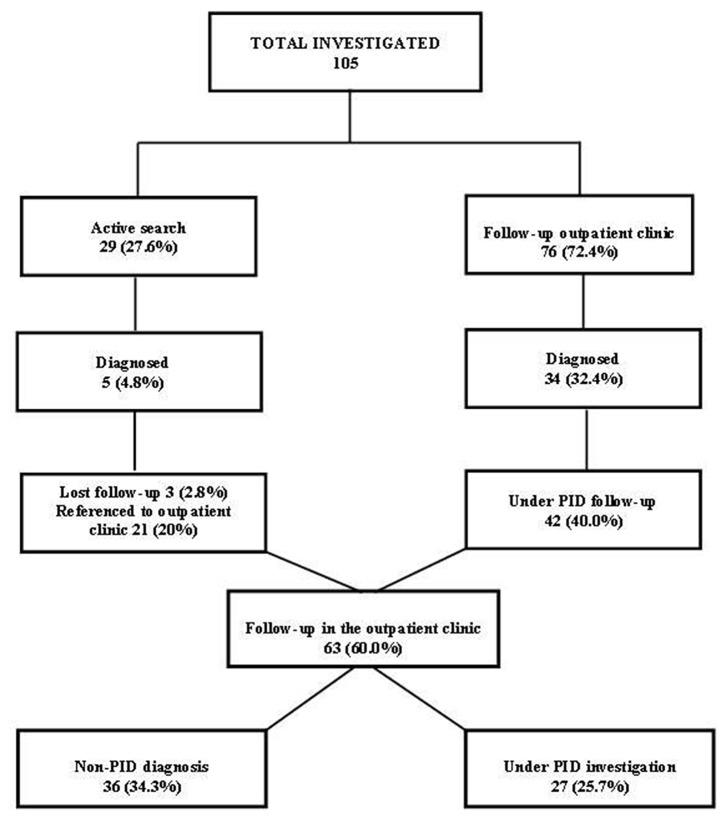
Flow chart of patients investigated and followed up in the Regional Hospital of Presidente Prudente, 2014–2018.

Over 5 years of follow-up (2014–2018) for immunologic evaluation and management, 105 patients were investigated as follows: (1) between January 2014 and December 2018, a retrospective study was conducted on 76 individuals referred by other clinics; (2) between September 2017 and December 2018, a prospective study investigated 29 patients identified by active search who attended weekly visits to the pediatric and infectious diseases wards and pediatric intensive care unit of RH (Figure 3).

The active search was conducted for patients with warning signs for PIDs proposed by the Jeffrey Modell Foundation (JMF) for children and adults. When at least one of the 10 signs for PID was found, the patient underwent directed anamnesis and a detailed physical examination and, if appropriate, initial laboratory screening for PIDs was performed. However, although globally used for screening PIDs, the JFM warning signs do not properly cover the non-infectious signs of PID. After hospital discharge, these patients were referred to the Immunodeficiency Outpatient Clinic of RH for follow-up (Figure 3). Patients for whom a diagnosis was not possible or who required immediate follow-up for genetic or immunologic complex tests were referred to the ambulatory unit for dermatologic manifestations of PIDs of the Hospital das Clínicas da Faculdade de Medicina da Universidade de São Paulo, São Paulo, Brazil, a reference center of immunology for RH. The diagnosis was established according to the Working Definitions for Clinical Diagnosis of PID of the European Society of Immunodeficiencies (ESID-2019) (8).

Informed consent was obtained from each patient's parent or guardian before enrollment in the study. Data were collected from patients' medical records, and their demographic characteristics (age, gender, age at diagnosis, antimicrobials used, and the number of hospitalizations), diagnosis of PIDs, recurrent infectious outbreaks, associated comorbidities, and non-infectious complications, which corroborated the classification of PID, were collected to generate a profile of these patients. Data on the spectrum of PID patients, complications in various systems, the characteristics of patients available for active search vs. patients referred to the outpatient clinic for primary immunodeficiency in RH, patients under investigation for PID, and non-PID diagnosis were recorded.

The initial screening for PIDs was performed by the following laboratory tests: complete blood cell counts, serum protein electrophoresis, total immunoglobulins A, E, G, and M, complement (C)3, C4, and CH50 (the hemolytic activity of the classic complement pathway). Specific antibody responses to pneumococcal polysaccharide vaccine (representing a T cell-independent response) and tetanus, diphtheria, and hepatitis B virus after vaccination (representing a T cell-dependent response) were investigated. Immunophenotyping and lymphocyte proliferation were performed in a limited number of patients. The Kaplan–Meier estimator was used to estimate the survival probability.

In the active search, all patients with a diagnosis of PID confirmed by other services and those with other defined diagnoses that could interfere with the classification of PID were excluded, even if they presented some warning signs.

### Geospatial Analysis and Construction of Maps

For geospatial analysis, maps were constructed using geographic information system (GIS) technology and spatial statistics. In spatial statistics, the study of punctual processes was explored, focusing on the spatial distribution of the residence of patients by a municipality in RHAN11. In the GIS technology, ArcGIS module visualization was used as developed by the Environmental Systems Research Institute (ArcGIS software 10.2.2; ESRI, Redlands, CA). Both processes transcribe the information from the database to the maps, giving a broad view of the phenomena and their concentrations. The geographic distribution of PID network centers in Brazil and the number of registered patients in 2018 were obtained from the Latin American Society for Immunodeficiencies (LASID). The municipality of residence of patients was obtained from file records to investigate the geospatial distribution in the RHAN11 mesoregion. The cumulative incidence of PID (per 10,000 inhabitants) for the entire RHAN11 and for each municipality was determined. The urban area of Presidente Prudente municipality is highlighted in green in Figure 2, and municipalities with a population higher than 40,000 inhabitants in 2018 are marked with an asterisk.

### Statistical Analysis

The results are shown as means ± standard error of the mean (SEM) (for normally distributed variables). Dichotomous and nominal variables are expressed as frequencies and percentages. The survival rate was evaluated using the Kaplan–Meier method. The patients' municipality of residence was obtained from file records to investigate the geospatial distribution in the RHAN11 mesoregion. The incidence rates were the total number of patients diagnosed with PID for each municipality divided by the total population, per 10,000 inhabitants, during the period from 2014 to 2018. The incidence rate for RHAN11 was the total number of patients diagnosed with PID divided by the entire population, per 10,000 inhabitants, during the same period. Statistical analysis was performed using GraphPad Software (San Diego, CA, USA) and the Sigma-Stat program (Systat Software, Richmond, CA, USA).

## Results

### Epidemiologic Characteristics

Between January 2014 and December 2018, 105 patients were investigated for PID at the Regional Hospital of Presidente Prudente (Figure 3), distributed as follows: 76 patients (72.4%) were referred from other clinics to the outpatient immunodeficiency clinic at RH; 34 (32.4%) were diagnosed with a recognizable PID and 42 (40.0%) remained under investigation. Between September 2017 and December 2018, 29 (27.6%) were identified in an active search for patients with warning signs for PIDs in the pediatric and infectious diseases wards and pediatric intensive care unit of RH, and 5 (4.8%) were diagnosed with a recognizable PID. After discharge, 3 (2.8%) were lost follow-up and 21 (20%) were referred to the outpatient immunodeficiency clinic and remained under investigation. In December 2018, of 63 patients, 36 (34.2%) were classified with a non-PID diagnosis (Table S1), and 27 (25.7%) remained under investigation, waiting for more specific genetic tests.

**Figure 4:**
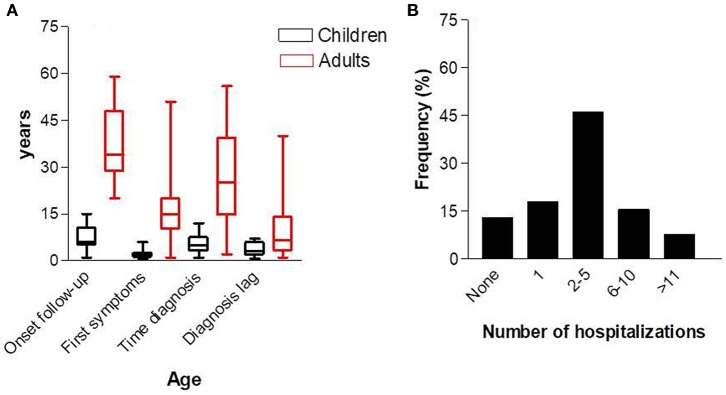
Age at first consultation, age of first symptoms, and diagnosis **(A)**, and number of hospitalizations **(B)**.

Among the 39 patients classified with PID, 23 (58.9%) were female and 16 (41.0%) were male with a ratio of 1.4:1. With regard to age, 19 (48.7%) patients were <17 years of age. The mean age of the whole group was 22.8 ± 17.7 years (IQR, 17.0–28.5 years; varying from 1 to 59 years). The mean age of the children was 7.3 ± 4.0 years (IQR, 5.3–9.2 years; varying from 1 to 15 years). The mean age of the adults was 37.5 ± 12.1 years (IQR, 31.8–43.1 years; varying from 20 to 59 years). The age at the first symptoms in the children was 2.0 ± 1.7 years (IQR, 1.1–2.8 years; varying from 6 months to 6 years), and in adults was 16.3 ± 11.8 years (IQR, 10.8–21.9 years; varying from 6 months to 51 years). The age at diagnosis in the children was 5.1 ± 3.1 (IQR, 3.6–6.6; varying from 1 to 12 years), and in adults was 27.1 ± 16.3 (IQR, 19.5–34.8; varying from 2 to 56 years). The diagnosis lag, which represents the time elapsed between onset of symptoms and diagnosis in the whole population, was 7.3 ± 8.6 years (IQR, 4.5–10.2 years; varying from 6 months to 40 years). The delay in diagnosis in children was 3.7 ± 2.1 years (IQR, 2.6–4.7), and in adults was 10.8 ± 10.9 years (IQR, 5.7–15.9 years; Figure 4A). During the 5 years of follow-up, 18 of 39 patients (46.1%) were hospitalized 2–5 times, and 3 of 39 patients (7.7%) were hospitalized more than 10 times (Figure 4B). To investigate the effect of PID care in the region during the 5 year follow-up, we compared the number of hospitalizations before and after diagnosis. The number of hospitalizations before the diagnosis was 3.7 ± 3.0 (IQR, 2.7–4.8) and the number after diagnosis was 1.5 ± 2.3 (IQR, 0.6–2.3).

### Clinical Characteristics

#### Spectrum of PID

**Figure 5:**
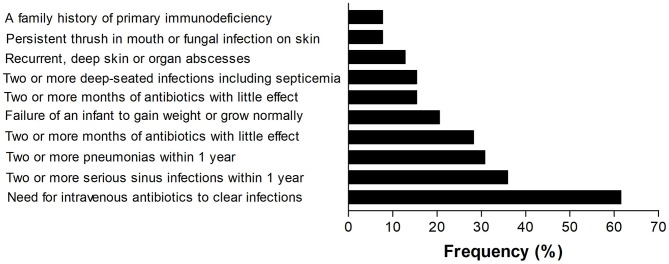
Frequency of the warning signs in 39 patients with PIDs.

Among the 39 patients (Table 1), antibody deficiency disease was the most common category and common variable immunodeficiency (CVID) was the most prevalent phenotype. All the patients with CVID were older than 4 years and the mean B lymphocyte count on immunophenotyping was 104.1 ± 124.8 (IQR, 0.2–208.5 cells/mm^3^). However, among the 10 classified patients, 5 (50%) were diagnosed in other PID reference centers before the creation of the outpatient immunodeficiency clinic in RH in January, 2014. The frequency of the 10 warning signs in our patients is shown in Figure 5. The need for intravenous antibiotics to clear infections and two or more serious sinus infections within 1 year were found in 24 (61.5%) and 14 (35.9%) patients, respectively. Among patients classified as predominantly antibody deficient, 15 of 22 (68.1%) presented with pneumonia at onset or during follow-up. Among the whole cohort, 4 (10.2%) patients did not show any warning signs.

**Table 1:** Clinical spectrum of PID.

**Immunodeficiency (*n* = 39)**	**Number**	**Clinical diagnosis/laboratory results**
Combined immunodeficiency	3	Reduced CD3 or CD4 or CD8 T cells; reduced proliferation to mitogens, severe infection requiring hospitalization
Hyper IgE syndrome (HIES)	3	IgE >10 times normal for age, pathologic susceptibility to infectious diseases with normal T and B lymphocyte counts
Ataxia telangiectasia (ATM)	1	Ataxia, oculocutaneous telangiectasia, and cerebellum hypoplasia on magnetic resonance imaging
Common variable immunodeficiency disorders (CVID)	10	Increased susceptibility to infections, marked decrease of IgG and marked decrease of IgA with or without low IgM levels and poor antibody response to vaccines
Deficiency of specific IgG (specific antibody deficiency [SPAD])	4	Infections (recurrent or severe bacterial) and normal serum/plasma IgG, A and M, and IgG subclass levels and profound alteration of the antibody responses to *Streptococcus pneumoniae* and normal serum/plasma IgG, A, and M, and IgG subclass levels and exclusion of T cell defect
Agammaglobulinemia	2	Fewer than 2% circulating B cells (CD19 and CD20), preferably in two separate determinations and a normal number of T cells (CD3, CD4, and CD8), absence of IgG levels
Selective IgA deficiency	4	Increased susceptibility to infection, and diagnosis after 4th year of life and undetectable serum IgA (when measured with nephelometry <0.07 g/L) but normal serum IgG and IgM (measured at least twice) and secondary causes of hypogammaglobulinemia have been excluded and normal IgG antibody response to all vaccinations and exclusion of T cell defect
Autoimmune lymphoproliferative syndrome (ALPS)	2	Splenomegaly, lymphadenopathy (>3 nodes, >3 months, non-infectious, non-malignant), vitamin B_12_ > 1,500 ng/L and IL-10 > 20 pg/mL
X-linked lymphoproliferative syndrome (XLP)	1	Male, hypogammaglobulinemia, and inflammatory bowel disease
Congenital neutropenia	1	Neutropenia below 0.5 g/L measured on more than three occasions, deep-seated infection due to bacteria and acquired community recurrent pneumonia, pyoderma gangrenosum, recurrent skin infections
Unclassified phagocytic disorders	1	Cystic fibrosis, respiratory infections, increased chloride in sweat
Hereditary angioedema (C1inh)	2	Recurrent angioedema without urticaria, recurrent abdominal pain, and family history of angioedema; low complement C4 and absent C1 esterase protein
Complement component 3 deficiency (C3)	1	Increased susceptibility to streptococcal infections, very low levels of C3
Chronic mucocutaneous candidiasis (CMC)	2	Chronic, persistent non-invasive mucocutaneous *Candida* infections (oral, esophageal, genital, skin, nails) confirmed with culture
Epidermodysplasia verruciformis	1	Extensive flat wart-like papules, usually on extremities, trunk and neck, innumerous basal cell, and squamous cell carcinomas

#### Complications in Various Organs

**Figure 6:**
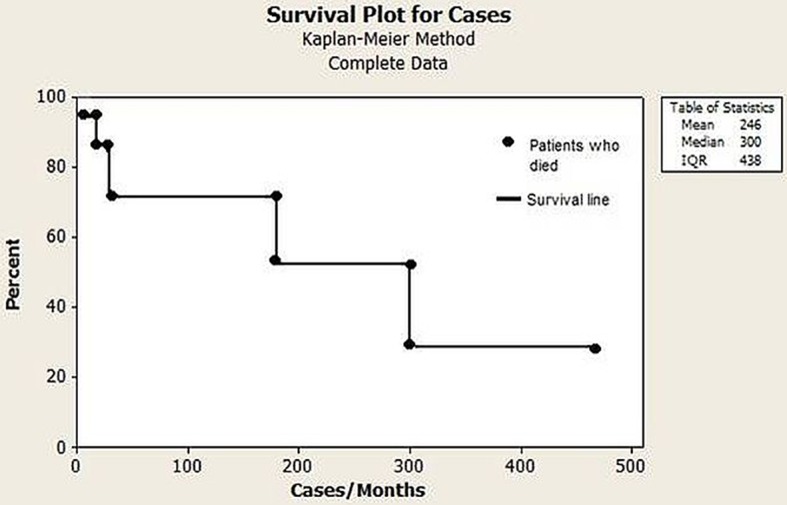
Kaplan–Meier survival curve of the 39 patients diagnosed with PIDs.

Autoimmune diseases accounted for most of the complications and were diagnosed in 24 (61.5%) patients. The most prevalent diseases were hemolytic anemia (20.5%), immune thrombocytopenia (10.2%), and Crohn disease (7.6%); 7.6% of the patients presented with asthma (Table 2). Six patients died (15.3%) during the study period. Of these patients, two were children who died at 2 and 3 years of age due to bronchopneumonia and respiratory failure, respectively. Their PID entities were combined immunodeficiency and specific antibody deficiency, respectively. The four adult patients who died were aged 35, 39, 54, and 55 years. Among the adults, fulminant infection and septicemia were the most common causes of death, and their PID entities were common variable immunodeficiency disorders (*n* = 2), pyoderma gangrenosum, and epidermodysplasia verruciformis (Figure 6). The percentage of consanguinity in the original population and the patient cohort was 0.95 and 2.5%, respectively.

**Table 2:** Complications of various organs during follow-up of 39 patients with primary immunodeficiency: 2014–2018.

**Type**	**Number (%)**
**Gastrointestinal system**	
Aphthous lesions	1 (2.5)
Celiac disease	2 (5.1)
Crohn disease	3 (7.6)
Malabsorption	6 (15.3)
Autoimmune hepatitis	2 (5.1)
Splenomegaly	9 (23.0)
Chronic diarrhea	9 (23.0)
Rectitis	1 (2.4)
**Renal system**	
Renal failure	1 (2.5)
Repeated urinary tract infection	2 (5.1)
Pyelonephritis	1 (2.5)
**Skin**	
Atopic dermatitis	4 (10.2)
Warts	2 (5.1)
Psoriasis	2 (5.1)
**Respiratory system**	
Bronchiectasis	3 (7.6)
Asthma	3 (7.6)
**Blood and lymph system**	
Hemolytic anemia	8 (20.5)
Lymphadenopathy	1 (2.5)
Immune thrombocytopenia	4 (10.2)
Lymphoma	1 (2.5)

#### Patients With Non-PID Diagnosis and Patients Under Investigation for PID

Among the 36 patients discharged as having a non-PID diagnosis, 16 different autoimmune diseases were identified, accounting for 68.0% of the patients. Atopic dermatitis was the most prevalent allergic disease (Table S1). The diagnosis of 26 patients is still under investigation. High levels of IgE were the most prevalent marker of a probable PID, accounting for 23.0% of the patients (Table S2). Pneumonia followed by diarrhea was the most prevalent illness, and lungs followed by skin were the most affected organs.

### Active Search

During the study, 29 patients in the pediatrics and infectious diseases wards and pediatric intensive care unit of RH were eligible for inclusion in the study due to the presence of one or more warning signs of PID. Most of the patients (62.0%) were aged <17 years. The mean age was 11.61 ± 2.35 years (IQR, 6.77–16.44 years; ranging from 1 to 47 years). Among those ≤17 years old, the mean age was 7.42 ± 1.03 years (IQR, 4.90–9.17 years), and 39.0 ± 2.97 years (IQR, 29.54–48.56 years) for those >18 years old. Five patients (12.8%) were diagnosed with PID, two with combined immunodeficiencies, one with autoimmune lymphoproliferative syndrome (ALPS), 1 with specific IgG deficiency (specific antibody deficiency), and one with hyper IgE syndrome.

Among the most prevalent infectious sites, 10 (34.4%) had upper respiratory tract infection, 8 (27.5%) had pneumonia, and 7 (24.1%) had cutaneous infections. After discharge, 21 patients (72.4%) attended the outpatient clinic for immunodeficiencies at RH to continue the investigation of PIDs. During the active search, two (7.1%) deaths occurred; one was due to severe pneumonia and sepsis and one was due to infection in the central nervous system and sepsis.

Table 3 shows the benefits of the active search from different aspects, such as early diagnosis, the involvement of multidisciplinary professionals, and dissemination of knowledge about PID, the warning signs, and how to screen eligible patients.

**Table 3:** Characteristics of patients available for active search vs. patients referred to the outpatient clinic for primary immunodeficiency in RH.

**Active search**	**Patients referred**
Early diagnosis and immediate intervention	Late diagnosis and intervention rarely immediate
Continuing medical education	Insufficient knowledge of professionals about warning signs
Higher impact on quality of life and mortality rates	Lower impact on quality of life and mortality rates
Lower number of hospitalizations and less antibiotics use	Increased number of hospitalizations and necessity for antibiotics
Lower prevalence of associated comorbidities	Late diagnosis is associated with higher prevalence of associated comorbidities

### Geographic Distribution of Patients and PID Network Centers in Brazil

Figure 1 shows that the number and geographic distribution of PID centers in Brazil is heterogeneous and differ greatly between the regions. According to LASID, most of the centers are located in the south and southeastern regions, few centers are located in the northeast, and none are located in the large northern region to date. Circles represent the number of patients diagnosed in 2018. Small centers may have documented only one patient, whereas the largest center in São Paulo documented 551 patients.

In the geographic distribution and cumulative incidence for patients with a PID diagnosis in the counties of the mesoregion of RHAN, Presidente Prudente, the main city in the region where RH is located, has the highest number of patients and an incidence of 0.67–2.43 per 10,000 inhabitants. The highest incidences were found in municipalities with a low number of inhabitants (2.43–4.09 per 10,000 inhabitants). Considering the overall population living in the 45 municipalities of RHAN, the cumulative incidence was 0.59 per 10,000 inhabitants. Three municipalities have a population higher than 40,000 inhabitants and heterogeneous distribution of patients in the domain of RHAN was found (Figure 2).

## Discussion

This study reports the 5 year experience of PID classification in a public, tertiary, university reference center located in an inner-city far from the PID network that covers a large mesoregion in São Paulo State. During this period, 105 patients were analyzed, and 39 patients were diagnosed with PID.

Regarding the epidemiologic characteristics, the female-to-male ratio was 1.4:1. The ratio varied according to the characteristics of the study population, reference centers, or country registers. Normally, males were more prevalent than females (9–12). The number of patients ≤17 years old was similar to the number of adults. In those ≤17 years old, the age of first symptoms was 2.0 ± 1.7 years and the diagnosis lag was 3.7 ± 2.1 years; in adults, the age of first symptoms was 16.3 ± 11.8 years and the diagnosis lag was 10.8 ± 10.9 years. These data show clearly that the delay in diagnosis in adults was three-fold higher than in patients ≤17 years old. One reason for this is that, in our region, pediatricians may be better prepared to recognize the warning signs for PID than general medical doctors. Since the creation of the outpatient immunodeficiency clinic in RH in 2014, pediatricians and pediatric residents have undergone continuing education, and in 2019, a workshop on “PIDs diagnosis: an approach to the non-immunologist” was conducted in RH with 42 participants. Furthermore, in Brazil, public health care programs for children are more efficient than for adults, increasing the possibility of PID diagnoses (13). The diagnosis of PID was key in reducing the number of hospitalizations during the 5 year follow-up period, from 3.7 to 1.5 times. As outlined in other studies, the diagnosis of PID has been shown to be cost-effective in preventing hospitalizations and the need for intravenous antibiotics to clear infections (2, 9, 10).

Patients classified as having predominantly antibody deficiencies represented 51.3% of the entire population analyzed. CVID followed by selective IgA deficiency, deficiency of specific IgG antibody and agammaglobulinemia were the prevalent phenotypes. These data are strongly supported by previous reports not only from registries in Latin American countries (6) and Europe (14) but also from reference center hospitals in Cairo, Egypt (9), and Guanajuato, Mexico (10). In Brazil, current data on PID characteristics are scarce. In 1997, in the Children's Hospital, University of São Paulo, a public, university, tertiary hospital, 166 children were classified phenotypically (15). In 2013, a report on patients with PID in the Hospital das Clínicas da Faculdade de Medicina da Universidade de São Paulo presented 1,008 cases of children and adults followed in the last 30 years. Antibody deficiencies were the most frequent disorders and IgA deficiency was the most prevalent phenotype, followed by transient hypogammaglobulinemia of infancy (16). In Latin American countries, in December 2018 according to the LASID database, selective IgA deficiency was the most prevalent disorder found, followed by CVID, transient hypogammaglobulinemia of infancy, specific antibody deficiency with normal IgG levels and normal B cells (17). We suggest that these differences are due to the fact that the spectrum of PID depends on the characteristics of the population analyzed as well as the laboratory resources available for molecular diagnosis. In our results, 40% of patients on intravenous immunoglobulin (IVIG) replacement were diagnosed before 2014 in other PID reference centers. IVIG was administered in an irregular manner due to difficulties with monthly treatments, including long distances (540 km from São Paulo), high prices for bus tickets, hotel stays, and the need for at least 3 working days. The creation of the PID ambulatory center in RH was the cornerstone for regularizing IVIG replacement and follow-up of these patients. Autoimmune diseases accounted for 61.5% of patients diagnosed, supporting the association between autoimmune diseases and PID, based on the concept that excessive inflammatory responses and autoimmunity are also common manifestations of immunodeficiency (4). Furthermore, in this study, among the patients discharged as having a non-PID diagnosis, autoimmunity was the most prevalent phenotype. One possible reason for increased rates of autoimmunity is that patients are referred to the outpatient immunodeficiency clinic mainly from dermatology, hematology, pediatrics gastroenterology, and rheumatology clinics. The association in patients seen in these services with autoimmune diseases is well-known (1, 4). In the first Brazilian report on PID carried out in a public reference hospital in São Paulo over an observation time of 15 years, autoimmune disorders were present in 5% of children (15). In an Italian cohort, 17.4% of patients had autoimmune manifestations before the diagnosis of CVID, and during a long-term follow-up, 25.9% had autoimmune diseases (18).

Regarding the 10 warning signs for PID, the need for intravenous antibiotics to clear infections, 2 or more serious sinus infections within 1 year and 2 or more pneumonias within 1 year were the most prevalent. From patients classified with predominantly antibody deficiencies, 68% presented pneumonia at onset or during follow-up. Respiratory infections are the most common presentations and the leading cause of morbidity and mortality in PIDs; those with CVID deserve special attention (18, 19). Furthermore, 46.1% of the patients with classified PIDs experienced 2–5 hospitalizations during the 5 years of follow-up, and the leading cause was respiratory infections. However, 7.6% of patients were hospitalized >11 times, illustrating the severity and suffering of these patients and their families. In this respect, 15.3% of these patients died due to bronchopneumonia and sepsis. These data are similar to the 12.7% found in a 15 year follow-up in a Brazilian report on PIDs in children (15), and are higher than the 6% mortality rates observed in Italian patients with CVID over a median follow-up of 11 years (18), as well as 13.0% observed in children with PIDs over 2 decades in a single center in Mexico (20). In registers, mortality rates may reach high levels, depending on the characteristics of the cohort. In the fourth update on the Iranian National Registry of PID, 43.0% of 3,056 patients died (11). As pointed out earlier, in our study, severe pneumonia and sepsis was the leading cause of death in 70.3% of the cases.

A new concept from this work was the benefit of the active search for patients with PID compared with passive demand, including early diagnosis, the involvement of multidisciplinary professionals and the dissemination of the knowledge about PID, the warning signs, and how to screen eligible patients. As far as we know, no data have been published on this issue in Brazil.

Although it is important for every Latin American country to determine the incidence and prevalence of PID in their respective countries, the actual frequency is unknown. With an estimated population of 753,344 inhabitants, the cumulative incidence of PID for the whole RHAN11 was 0.517 per 10,000 inhabitants, varying from 0.01to 4.09 per 10,000 inhabitants. The region is characterized by small- to medium-sized cities and few municipalities have a population higher than 40,000 inhabitants; municipalities with low numbers of inhabitants showed higher cumulative incidence. To our knowledge, these are the first data on the incidence of PID in São Paulo state. As expected, the municipality of Presidente Prudente had a high number of patients because its population represents 30.3% of the inhabitants of the mesoregion. However, some patients live a long distance from RH, highlighting the role of reference hospitals in the public health system in Brazil (21). The Jeffrey Model Foundation published an extensive collection of physician-reported prevalence for patients with PID around the world. In Latin America, there was an increase of 132.2% prevalence for patients followed and an increase in 71% of the reference centers for PIDs diagnosis in 2018 compared with 2013 (22). These data reinforce the importance and necessity of creating new centers for the diagnosis of PID in Brazil and elsewhere in Latin America.

Geoprocessing is an important tool for epidemiologic analysis due to its fast-updating power and precise identification of the spatial distribution of a studied population, however, there are few reports on the geographic distribution of patients with PIDs. In Mexico, most patients diagnosed with PIDs were concentrated in the cities around the capital, Mexico City (20). Brazil has a continental territory and different characteristics between regions. According to LASID, most of the PID network centers are concentrated in the southeastern region, particularly in São Paulo State, showing the asymmetry and the lack of public health policies in implementing new PID centers, mainly in the north and northeast region of Brazil. Supporting these data, about 50% of patients who attended the ambulatory center for dermatological manifestations of PIDs in the Clinics Hospital, São Paulo, SP, live in cities such as Porto Velho, Belém, and Manaus, 2.236, 2.879, and 3.872 km from São Paulo, respectively. In Germany, some regions have a high number of documenting PID centers and a large number of patients, whereas other small centers have documented only one patient (23).

Our results, obtained from patients attending an inner-city hospital in an asymmetric and poor region, with limited laboratory resources, may have global relevance mainly in developing countries. In Brazil, this study can be considered a model for poor-resource settings mainly in northeastern and northern regions for screening patients with PIDs, to improve the diagnosis and treatment and when necessary, to refer these patients to PID network centers for genetic diagnosis. Furthermore, in hospitalized patients, an active search for PIDs may be implemented.

### Limitations

Some shortcomings should be addressed when analyzing our data. Although RH is a university hospital, the lack of molecular diagnostics, a research laboratory, and funding for implementation of new techniques delay the diagnosis and the optimization of treatment in some cases. These patients have to be referred to network centers far from RH, which are also limited in their capacity to receive new patients and to perform molecular techniques. In the active search, the same warning signs were applied to children and adults, leading to selection bias. As a consequence, the efficacy of the selection may be affected. Furthermore, they do not cover a wide spectrum of PIDs in which infectious diseases are not present such as autoimmunity and malignancies (1, 4).

## Conclusions

Undoubtedly, the implementation of an outpatient immunodeficiency clinic in a referral hospital covering a mesoregion with a large population has led to the generation of policies and practices to improve the diagnosis, quality of life, and care of patients with PIDs and their families. Furthermore, the search among hospitalized patients for warning signs of PIDs showed great benefits. Great inequality in the distribution of PID network centers in Brazil was demonstrated.

## Data Availability Statement

The datasets for this manuscript are not publicly available due to agreements with the Regional Hospital of Presidente Prudente, São Paulo, Brazil. Requests to access the datasets should be directed to the RH via the corresponding author.

## Ethics Statement

The study was in accordance with the ethical standards of the Institutional Ethics Committee of Oeste Paulista University, Presidente Prudente, São Paulo, Brazil (number 4041/2017), and in keeping with the Helsinki Declaration of 1964, as revised in 1975, 1983, 1989, 1996, and 2000. Written informed consent to participate in this study was provided by the participants' legal guardian/next of kin.

## Author Contributions

Conceptualization: LP-C and DM-V. Follow-up of the patients and clinical data collection: DB, LP, GP, and LP-C. Geospatial analysis and construction of the maps: EF and AC-N. Writing—original draft: LP-C. Writing—review and editing: DB, LP, GP, EF, DM-V, AC-N, and LP-C.

## Conflict of Interest

The authors declare that the research was conducted in the absence of any commercial or financial relationships that could be construed as a potential conflict of interest. The handling editor declared a past collaboration with one of the authors AC-N.
