# Supplementary material for: Primary Immunodeficiencies in a Mesoregion of São Paulo, Brazil: Epidemiologic, Clinical, and Geospatial Approach
Source: Front Immunol. 2020 May 12;11:862. doi: 10.3389/fimmu.2020.00862 (PMC7235164; doi:10.3389/fimmu.2020.00862)
Supplement: Supplementary file 1 [file Data_Sheet_1.docx]

**TABLE S1** | Non-PID diagnosis (*n* = 36).

| Diagnosis | Percentage |
| --- | --- |
| Autoimmune diseases |  |
| Atopic dermatitis | 12.10 |
| Asthma | 9.75 |
| Type II diabetes mellitus | 7.31 |
| Inflammatory bowel disease | 4.87 |
| Autoimmune hepatitis | 4.87 |
| Human papillomavirus | 4.87 |
| Chronic kidney disease | 2.43 |
| Glaucoma | 2.43 |
| Thrombocytopenic purpura | 2.43 |
| Fibromyalgia | 2.43 |
| Anemia | 2.43 |
| Kikuchi syndrome | 2.43 |
| Peripheral neuropathy | 2.43 |
| Autoimmune uveitis | 2.43 |
| Hashimoto-Graves syndrome | 2.43 |
| Myasthenia gravis | 2.43 |
| Kaposi sarcoma | 4.87 |
| IgE elevation angioedema | 4.87 |
| Erythema multiforme | 4.87 |
| Syphilis | 2.43 |
| Toxoplasmosis | 2.43 |
| Sickle cell anemia | 2.43 |
| Systemic lupus erythematosus | 2.43 |
| Dengue | 2.43 |
| Myelodysplasia | 2.43 |
| Omim 24 syndrome | 2.43 |

**TABLE S2 |** Spectrum of 26 patients under investigation for PID.

| **Probable PID** | **No. (%)** | **Illnesses** | **Organs** |
| --- | --- | --- | --- |
| Hyper IgE | 6 (23.0) | Diarrhea, recurrent pneumonia, sinusitis, otitis, tonsillitis | Lungs, skin, upper airways |
| Deficiency of specific immunoglobulins | 5 (19.2) | Abscess, diarrhea, pneumonia, tonsillitis, otitis | Gastrointestinal tract, lungs, upper airways |
| Complement deficiency | 4 (15.4) | Pneumonia, sinusitis, telangiectasis | Lungs, skin, upper airways |
| Chronic granulomatous disease | 3 (11.5) | Herpes, Cytomegalovirus, Epstein-Barr virus infections | Lungs, upper airways |
| Autoimmune lymphoproliferative syndrome | 2 (7.6) | Pneumonia, pyoderma | Lymphadenopathy |
| Unclassified phagocytic disorders | 2 (7.6) | Tonsillitis, abscess, fungal infections, sepsis | Lungs, skin |
| Combined immunodeficiency | 2 (7.6) | Latent tuberculosis | Lungs, skin, upper airways |
| Defects with susceptibility to *Mycobacterium* species | 1 (3.8) | Myositis, diarrhea, Crohn disease | Gastrointestinal tract, bladder, lymph nodes |
| Early-onset multi-organ autoimmune disease | 1 (3.8) | Pneumonia, tonsillitis, atopic dermatitis, fungal infections, asthma, chronic furunculosis | Muscles, gastrointestinal tract |
